# Kinetics of Microbial Translocation Markers in Patients on Efavirenz or Lopinavir/r Based Antiretroviral Therapy

**DOI:** 10.1371/journal.pone.0055038

**Published:** 2013-01-28

**Authors:** Jan Vesterbacka, Piotr Nowak, Babilonia Barqasho, Samir Abdurahman, Jessica Nyström, Staffan Nilsson, Hiroyuki Funaoka, Tatsuo Kanda, Lars-Magnus Andersson, Magnus Gisslèn, Anders Sönnerborg

**Affiliations:** 1 Unit of Infectious Diseases, Department of Medicine, Karolinska Institutet, Karolinska University Hospital, Huddinge, Stockholm, Sweden; 2 Department of Laboratory Medicine, Division of Clinical Microbiology, Karolinska Institutet, Karolinska University Hospital, Huddinge, Stockholm, Sweden; 3 Department of Mathematical Statistics, Chalmers University of Technology, Gothenburg, Sweden; 4 DS Pharma Biomedical Company, Limited, Osaka, Japan; 5 Division of Digestive and General Surgery, Niigata University Graduate School of Medical and Dental Science, Niigata, Japan; 6 Department of Infectious Diseases, University of Gothenburg, Gothenburg, Sweden; Rush University, United States of America

## Abstract

**Objectives:**

We investigated whether there are differences in the effects on microbial translocation (MT) and enterocyte damage by different antiretroviral therapy (ART) regimens after 1.5 years and whether antibiotic use has impact on MT. In a randomized clinical trial (NCT01445223) on first line ART, patients started either lopinavir/r (LPV/r) (n = 34) or efavirenz (EFV) containing ART (n = 37). Lipopolysaccharide (LPS), sCD14, anti-flagellin antibodies and intestinal fatty acid binding protein (I-FABP) levels were determined in plasma at baseline (BL) and week 72 (w72).

**Results:**

The levels of LPS and sCD14 were reduced from BL to w72 (157.5 pg/ml vs. 140.0 pg/ml, p = 0.0003; 3.13 ug/ml vs. 2.85 ug/ml, p = 0.005, respectively). The levels of anti-flagellin antibodies had decreased at w72 (0.35 vs 0.31 [OD]; p<0.0004), although significantly only in the LPV/r arm. I-FABP levels increased at w72 (2.26 ng/ml vs 3.13 ng/ml; p<0.0001), although significantly in EFV treated patients only. Patients given antibiotics at BL had lower sCD14 levels at w72 as revealed by ANCOVA compared to those who did not receive (Δ = −0.47 µg/ml; p = 0.015).

**Conclusions:**

Markers of MT and enterocyte damage are elevated in untreated HIV-1 infected patients. Long-term ART reduces the levels, except for I-FABP which role as a marker of MT is questionable in ART-experienced patients. Why the enterocyte damage seems to persist remains to be established. Also antibiotic usage may influence the kinetics of the markers of MT.

**Trial Registration:**

ClinicalTrials.gov NCT01445223

## Introduction

A sustained control of the human immunodeficiency virus type 1 (HIV-1) replication is obtained by antiretroviral therapy (ART) in the majority of patients, reducing plasma HIV-1 load to undetectable levels. However, HIV-1 persists in reservoirs like latently infected CD4+ T cells [1], [2] and in body compartments that have restricted permission for antiretroviral drugs. It is hypothesized that these reservoirs are refilled by the low grade viral replication seen in patients on suppressive ART who otherwise have undetectable viremia by routine assays [3], [4]. Translocation of bacterial products across a damaged gut-blood barrier has been proposed to be one important mechanism for the persistence of a chronic immune activation which is found also in well-treated patients [5], [6]. There is growing evidence that this immune activation may contribute to the low-grade viremia and e.g. cardiovascular and CNS complications [7], [8], [9].

Several markers are used to assess microbial translocation (MT) in patients with HIV or inflammatory bowel disease [10], such as microbial products [lipopolysaccharide (LPS), plasma bacterial 16SrDNA, anti-flagellin antibodies] [11], markers of the systemic response to bacterial products (sCD14, LPS-binding protein), and of enterocyte damage [intestinal fatty acid binding protein (I-FABP)] [12], [13]. During successful ART, MT and systemic immune activation are usually reduced, but not normalized, suggesting that the damage of the gut-blood barrier is only partly restored [5]. The reasons why this improvement varies between patients are not known.

The origin of low-level viremia in patients on suppressive ART has been disputed. Residual virus replication from anatomical compartments like the gut could be one of the explanations. Firstly, levels of HIV-1 DNA and RNA were substantially elevated in the gut compared with peripheral blood in patients on ART with <40 HIV-1 RNA copies/ml, indicating that the gut may serve as a potential source of viremia during suppressive ART [14]. Additionally, in a set of patients on long-term ART, levels of HIV DNA in sigmoid colon were positively correlated to plasma LPS levels [15], suggesting a connection between residual viremia and MT. In a cross-sectional study of patients with persistently undetectable HIV-1 RNA, a higher proportion of participants treated with nevirapine and efavirenz achieved <2.5 HIV-1 RNA copies/ml compared to lopinavir/r based ART [16]. Given the link between MT and low-level viremia, we assumed that choice of ART could differently affect kinetics of MT markers.

In the present study, we analyzed the levels of LPS, sCD14, I-FABP, and anti-flagellin antibodies, at baseline (BL) and after 72 weeks (w72) of ART in a controlled randomized clinical trial in which the patients received either lopinavir/r +2 nucleoside analogues (NRTI) or efavirenz +2 NRTI. Additionally, we studied if ongoing antibiotics treatment had an impact on the explored parameters.

## Methods

### Subjects

**Figure 1:**
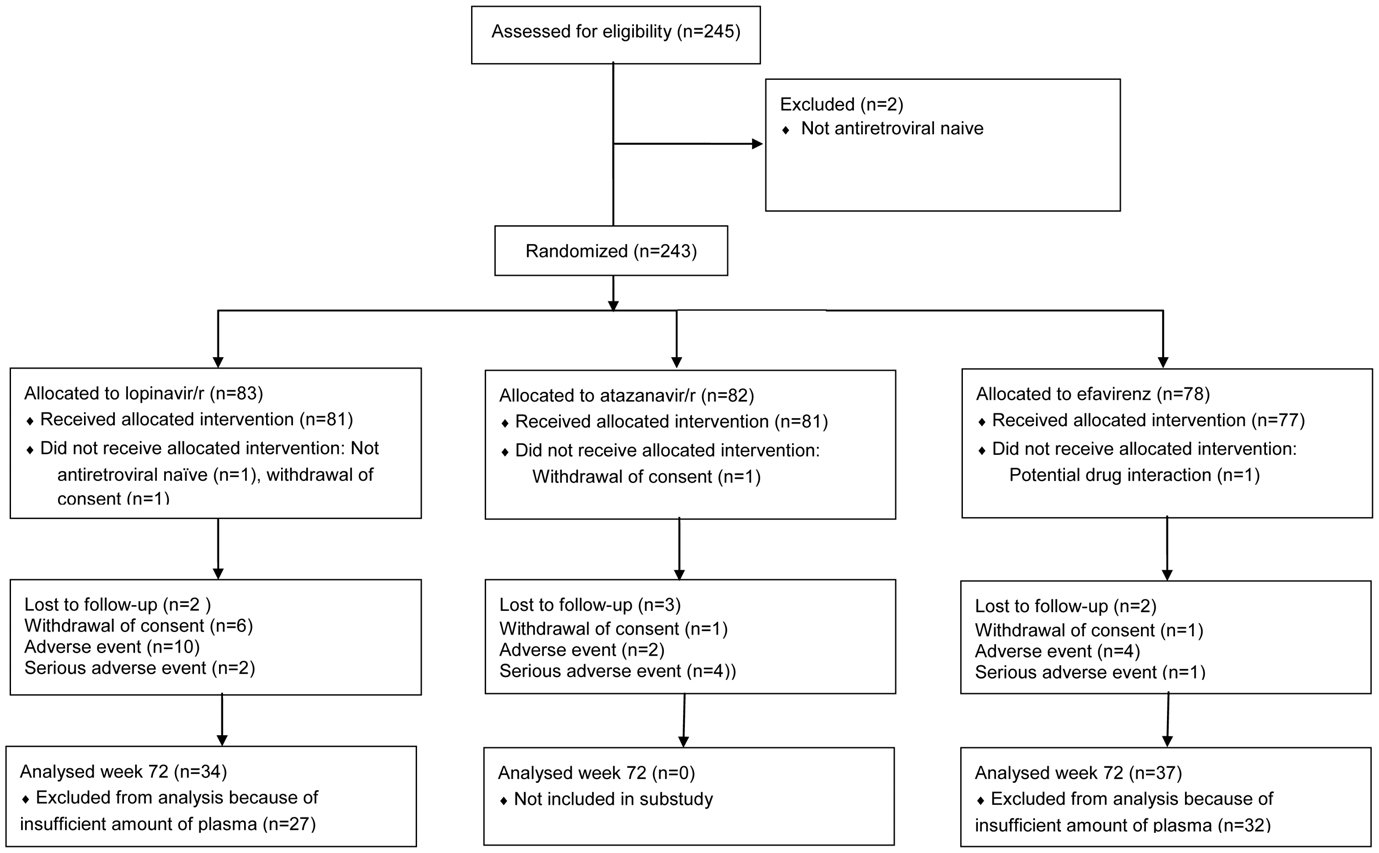
Trial profile and reasons for discontinuations in each group.

During 2004–2007, 239 HIV-1 infected subjects received allocated intervention after written consent in a Scandinavian randomized clinical phase IV efficacy trial (RCT) (ClinicalTrials.gov identifier: NCT01445223). The protocol for this trial and supporting CONSORT checklist are available as supporting information; see Checklist S1 and Protocol S1. The study protocol was approved by the Regional Ethics Committee (Gothenburg Ö739-03). The study design and participants have been described elsewhere [17], [18]. In our substudy, the patients were randomized to receive either efavirenz (EFV) +2 NRTI once daily (n = 37) or ritonavir-boosted lopinavir (LPV/r) +2 NRTI twice daily (n = 34) (Figure 1). Totally 59 patients were excluded from our analysis because of insufficient remaining plasma volumes after the main study analyzes. CD4+ T-cell count, viral load (VL), rate of hepatitis B/C co-infection and age were similar in the group of excluded patients at BL, compared to the substudy group.

Data on antibiotic therapy was available in 63 patients of whom 29 were given antibiotics at baseline (BL) (n = 27) and/or week72 (w72) (n = 10), while 34 had not received antibiotics at any of the two time points (Table 1). At BL, the patients received: cotrimoxazole (TMP-SMX) as *Pneumocystis jiroveci* prophylaxis (PCP) (n = 24) or for treatment of pneumonia (n = 2), or clindamycin (n = 1). Also, two were on *Mycobacterium tuberculosis* treatment, and one was on fluconazole. At w72, TMP-SMX was given as PCP prophylaxis (n = 9), and one patient received nitrofurantoin; 8 of these 10 had had TMP-SMX at baseline.

**Table 1:** Baseline characteristics of antibiotic treated patients.

	All	Antibiotic treated	Other
**Subjects [number]**	63	27	36
**CD4+ count/ul [median (range)]**	160 (90–205)	120 (30–150)	180 (138–220)
**HIV-1 RNA [median log10 copies/ml (range)]**	5.25 (2.95–7.07)	5.34 (3.86–7.07)	5.15 (2.95–6.66)

### CD4^+^ T-cells and Plasma HIV-1 RNA

HIV RNA load and CD4^+^ T-cell counts were measured as part of the clinical routine with Cobas Amplicor (Roche Molecular Systems Inc., Branchburg, New Jersey, USA), and flow cytometry, respectively.

### Microbial Translocation Markers

Plasma samples obtained at the sampling day were frozen at −80°C and later thawed. The analyses were performed blindly in relation to clinical data and treatment outcome. Levels of LPS were measured by limulus amebocyte assay (LAL, Lonza, Maryland, USA) as previously described [5]. sCD14 and I-FABP levels were determined by enzyme-linked immunosorbent assays (R&D Systems, USA and DS Pharma Biomedical Co, Japan; respectively), according to the manufactureŕs instructions [19]. Samples at BL and w72 from the same patient were assayed on the same plate.

Antibody titers to flagellin, and total IgG levels were assessed by an in-house anti-flagellin specific IgG ELISA [20] using purified flagellin monomers from *S. typhimurium* (InvivoGen, USA). It is known that human sera have a similar recognition pattern of flagellin monomers whether isolated from flagellated *E. coli* or *S. typhimurium* [21]. Briefly, microwell plates (MWP) were coated overnight with purified flagellin from *S. typhimurium* (25 ng/well). The following day, plasma samples from HIV-1 patients were diluted 1∶1000 and applied to the MWP. After incubation and washing, the MWPs were incubated with HRP-conjugated anti-human IgG. For total IgG ELISA, the manufacturer’s procedure was followed (MABTECH, Nacka, Sweden).

### Statistical Analysis

Data were analyzed using GraphPad Prism v. 5.02 and R 2.13.1. Independent groups were compared using Mann-Whitney U-test and paired data analyzed with Wilcoxon signed rank test. Correlations were analyzed by Spearmańs rank test. Differences in LPS, sCD14, I-FABP and anti-flagellin IgG levels between patients with or without antibiotics were analyzed with ANCOVA using covariates age, sex, log viral load and CD4+ T-cell count at BL or w72, and residual plots were inspected. Model selection was done by backward elimination with removal of variables if P>0.1.

## Results

### HIV RNA Load and Recovery of CD4+ T-cells

Following initiation of ART, all patients achieved plasma HIV-RNA <50 copies/mL within 24 weeks, except for five patients (all <250 c/mL). Four of these reached undetectable HIV-RNA at w72. Two patients had a relapse with detectable HIV-RNA at w72 (190 respective 640 c/mL). No statistically significant differences were found at BL or follow-up between the two arms (Table 2). BL CD4+ T-cells in the LPV/r group (median 155 [IQR 119.5–203.3] cells/ul) and in the EFV group (130 [IQR 69–200] cells/ul) did not differ significantly (Table 2). There was no difference with regard to CD4+ T-cell recovery at w72 (LPV/r: 199 [IQR 135–294]; EFV: 156 [IQR 90–270]) or w48 (p = 0.27) (data not shown). At w72 the CD4+ T-cell count tended to be higher in the LPV/r group as compared to the EFV group (data not shown). There was no correlation between change in CD4+ T-cells and change of any of the markers between BL and w72.

**Table 2:** Characteristics of HIV-1 infected patients.

	All	Lopinavir-arm*	Efavirenz-arm*	
**Subjects [number]**	71	34	37	
**Age [years, median (range)]**	37 (19–70)	41 (19–68)	35 (20–70)	
**Sex [male/female]**	51/20	24/10	27/10	
**Hepatitis [B/C]**	1/3	1/2	0/1	
**CD4+ count/ul [median (range)]**				
** BL**	140 (0–370)	155 (11–370)	130 (0–270)	
** w72**	330 (73–1260)	370 (160–1260)		300 (73–843)
**HIV-1 RNA [median log10copies/ml (range)]**				
** BL**	5.3 (2.95–7.07)	5.3 (2.95–6.72)	5.3 (3.23–7.07)	
** w72**	0 (0–2.81)	0 (0–2.28)	0 (0–2.81)	
**Antibiotics use** 1				
** BL**	27	10	17	
** w72**	10	3	7	

* There were no statistically significant differences in age, CD4+ -cell count or HIV-1 RNA between the two treatment arms at BL. Abbreviations: BL, baseline.

1 Data from 63 subjects were available.

### Decrease of LPS and sCD14 after 72 Weeks of ART

**Figure 2:**
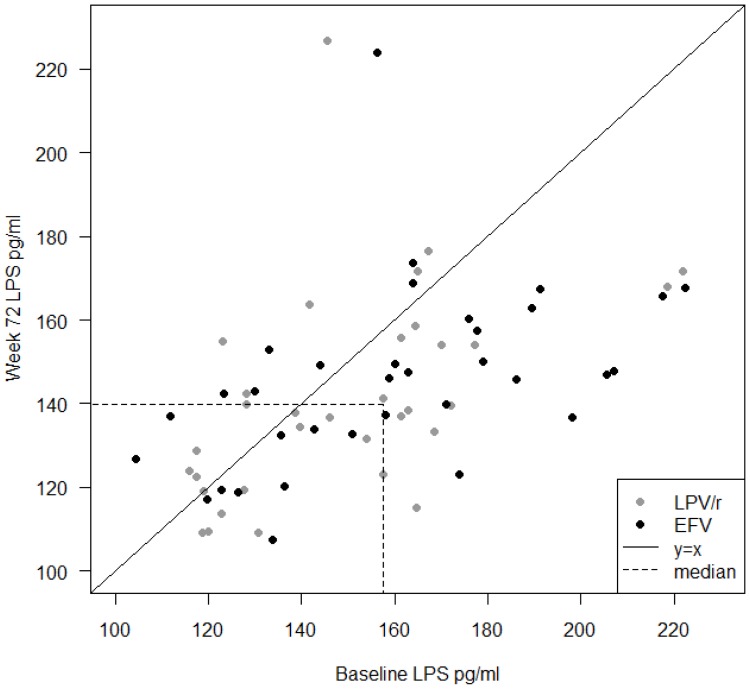
Levels of LPS. Levels of LPS were significantly reduced after 72 weeks of antiretroviral therapy in the whole cohort, with a unified pattern in both treatment arms. Data are shown as individual values with corresponding baseline LPS levels at X-axis and week 72 levels at Y-axis, respectively. Individuals treated with lopinavir (LPV/r) are indicated with shaded and efavirenz (EFV) with black circles. Dotted lines refer to median values at the corresponding time-point.

The overall plasma levels of LPS were reduced at w72 compared to BL (157.5 pg/ml [IQR 128.2–171.1] vs. 140.0 [IQR 127.8–155.2]; p = 0.0003) (Figure 2). The reduction in LPS levels and absolute change between BL and w72 was similar in both treatment arms (data not shown). No significant correlation was observed between LPS and CD4+ T-cell count, VL, sCD14 or I-FABP, respectively.

**Figure 3:**
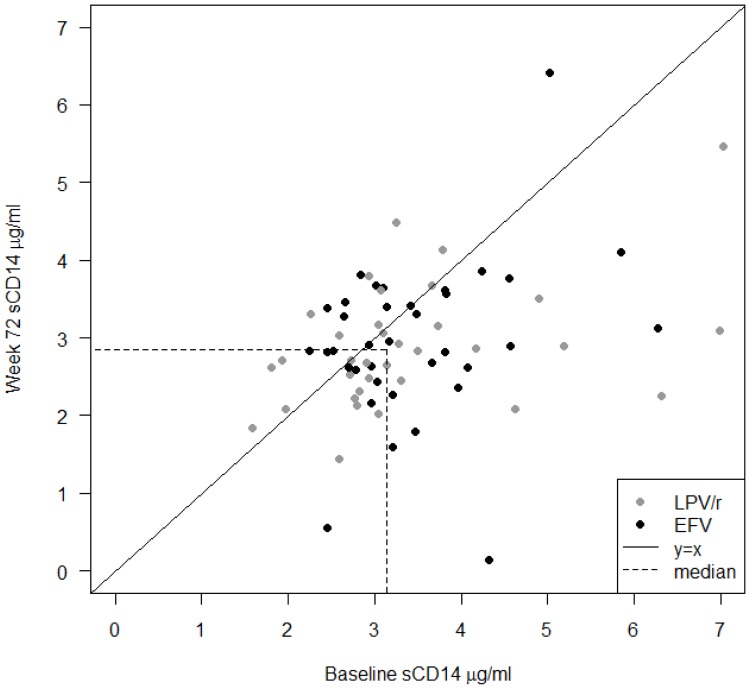
Levels of sCD14. Levels of sCD14 were significantly reduced after 72 weeks of antiretroviral therapy in the whole group, with a unified pattern in both treatment arms. Data are shown as individual values with corresponding baseline sCD14 levels at X-axis and week 72 levels at Y-axis, respectively. Individuals treated with lopinavir (LPV/r) are indicated with shaded and efavirenz (EFV) with black circles. Dotted lines refer to median values at the corresponding time-point.

**Figure 4:**
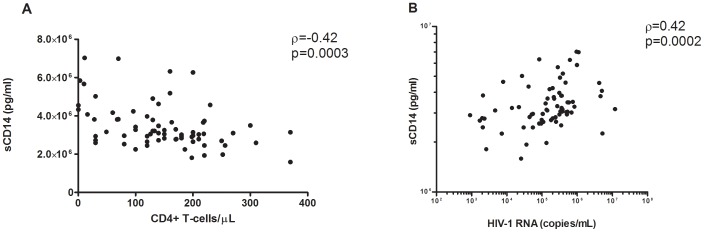
Correlations between sCD14, CD4+ T-cells and HIV-1 RNA. At baseline, significant correlations were found between sCD14 and CD4-T cell counts (**A**) or HIV-1 RNA viral load (**B**), respectively. Rho and p-value refer to Spearman’s rank test.

Similar to LPS, plasma levels of sCD14 were reduced at w72 compared to BL (3.13 ug/ml [IQR 2.7–3.8] vs. 2.85 ug/ml [IQR 2.4–3.4]; p = 0.005) (Figure 3) in both treatment arms. The CD4+ T-cell count and sCD14 levels showed a highly significant negative correlation at BL (ρ = −0.42, p = 0.0003) (Figure 4A) and at w72 (ρ = −0.32, p = 0.007). Viral load and sCD14 levels correlated at baseline (ρ = 0.42, p = 0.0002) (Figure 4B).

### Elevated Levels of I-FABP after 72 Weeks of EFV Containing ART

**Figure 5:**
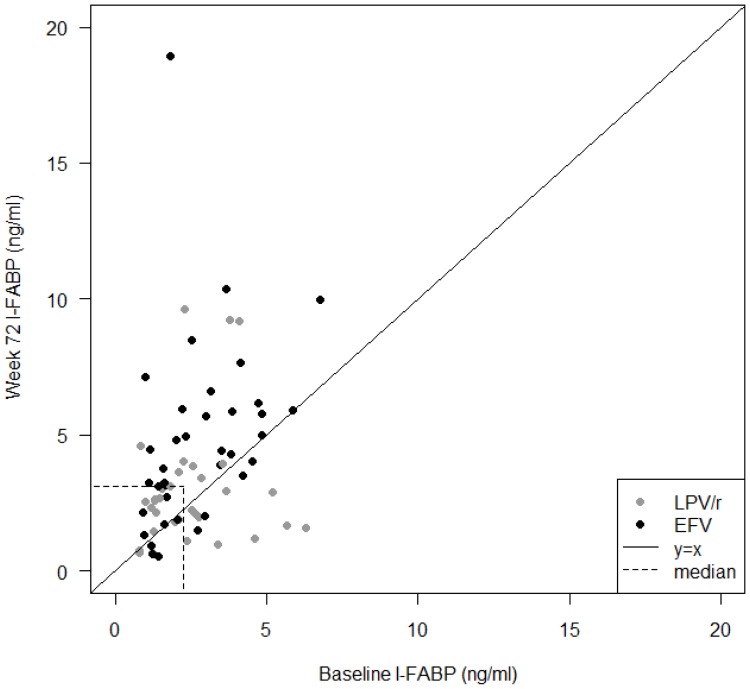
Levels of Intestinal Fatty Acid Binding Protein (I-FABP). Plasma levels of I-FABP were significantly elevated after 72 weeks of antiretroviral treatment as compared to baseline in the whole cohort. Stratified to treatment arms, efavirenz (EFV) treated patients had significantly increased levels**,** but not the patients on lopinavir/r (LPV/r) therapy. Data are shown as individual values with corresponding baseline I-FABP levels at X-axis and week 72 levels at Y-axis, respectively. Individuals treated with lopinavir (LPV/r) are indicated with shaded and efavirenz (EFV) with black circles. Dotted lines refer to median values at the corresponding time-point.

At BL, no difference was found in plasma I-FABP levels between the treatment groups. At w72, the overall I-FABP concentrations had increased (median 2.26 ng/ml [IQR 1.4–3.6] at BL vs 3.13 ng/ml [IQR 1.8–4.9] at w72; p<0.0001) (Figure 5). The elevation was significant only in the EFV arm (2.32 [IQR 1.5–3.8] vs. 4.29 [IQR 2.4–5.9]; p<0.0001), but not in the LPV/r arm (2.19 [IQR 1.3–3.4] vs. 2.56 [IQR 1.7–3.5]). The difference in elevation between the two arms was significant (p = 0.035). A significant difference was also found between the treatment arms at w72 (EFV: median 4.29 [IQR 2.4–5.9]; LPV/r: 2.56 [IQR 1.7–3.5]; p = 0.003). There was no significant correlation between I-FABP levels and CD4+ T-cells, VL, and sCD14, respectively.

### Decrease of Anti-flagellin Antibodies after 72 Weeks of ART

**Figure 6:**
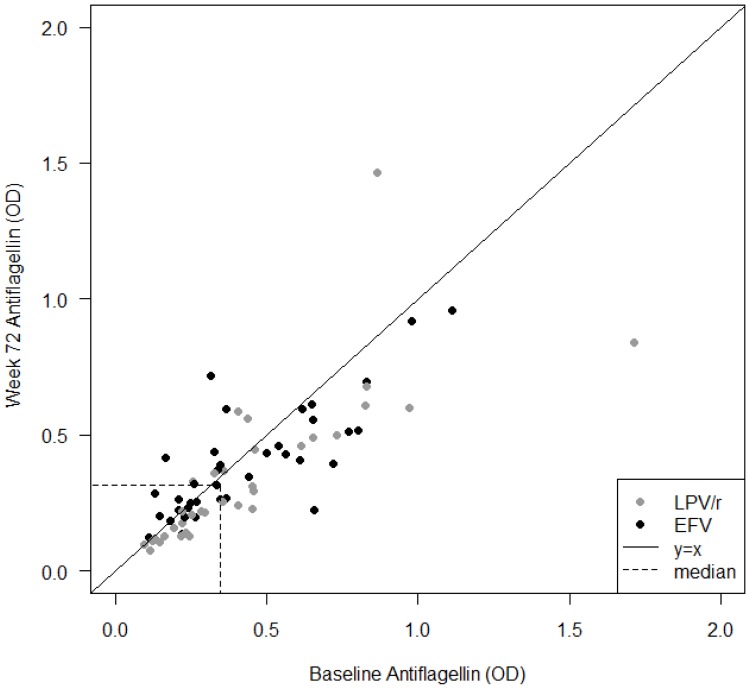
Plasma levels of anti-flagellin antibodies. A significant reduction of anti-flagellin IgG levels was seen after 72 weeks of antiretroviral treatment compared to baseline in the whole cohort. Plasma levels of anti-flagellin IgG decreased significantly in lopinavir/r (LPV/r) treated patients, but not during efavirenz (EFV) therapy. Data are shown as individual values with corresponding baseline anti-flagellin IgG levels at X-axis and week 72 levels at Y-axis, respectively. Individuals treated with lopinavir (LPV/r) are indicated with shaded and efavirenz (EFV) with black circles. Dotted lines refer to median values at the corresponding time-point.

Anti-flagellin antibodies were detected in all subjects. ART reduced the levels in the whole cohort (median OD 0.35 [IQR 0.22–0.61] at BL vs. 0.31 [IQR 0.21–0.5]) at w72; p = 0.0004) (Figure 6). In the LPV/r arm, anti-flagellin antibodies decreased significantly (median OD 0.34 [IQR 0.22–0.5] vs. 0.25 [IQR 0.14–0.49]; p = 0.001), but not in the EFV treated patients (median OD 0.35 [IQR 0.23–0.63] vs. 0.37 [IQR 0.24–0.51]; p = 0.06).

As expected, the total IgG concentrations were reduced at w72 (median OD 1.02 [IQR 0.99–1.07] at BL vs 0.99 [IQR 0.96–1.02] at w72; p = 0.0001) in the entire cohort, and in both treatment arms (LPV/r arm median OD 1.02 [IQR 0.99–1.05] at BL vs 0.98 [IQR 0.94–1.01] at w72; p<0.0001); (EFV arm median OD 1.03 [IQR 0.98–1.08] at BL vs 1 [IQR 0.96–1.03] at w72; p<0.0001). The anti-flagellin IgG:total IgG ratio was reduced between BL and w72 in whole cohort (median 0.34 [IQR 0.22–0.53] vs 0.3 [IQR 0.21–0.5]; p = 0.005) and LPV/r arm (median 0.34 [IQR 0.22–0.48] vs 0.26 [IQR 0.15–0.47]; p = 0.003), supporting that the decrease of anti-flagellin IgG was specific. This reduction was not observed in EFV treated (median 0.34 [IQR 0.23–0.59] vs 0.37 [IQR 0.25–0.52]; p = 0.31). LPS and anti-flagellin antibodies correlated at BL (ρ = 0.25, p = 0.04) and at w72 (ρ = 0.31, p = 0.01). On the contrary, there was no significant correlation at BL or w72 between anti-flagellin antibodies and CD4+ T-cells, VL, sCD14 or I-FABP, respectively.

### Antibiotic Treatment and Markers of Microbial Translocation

**Figure 7:**
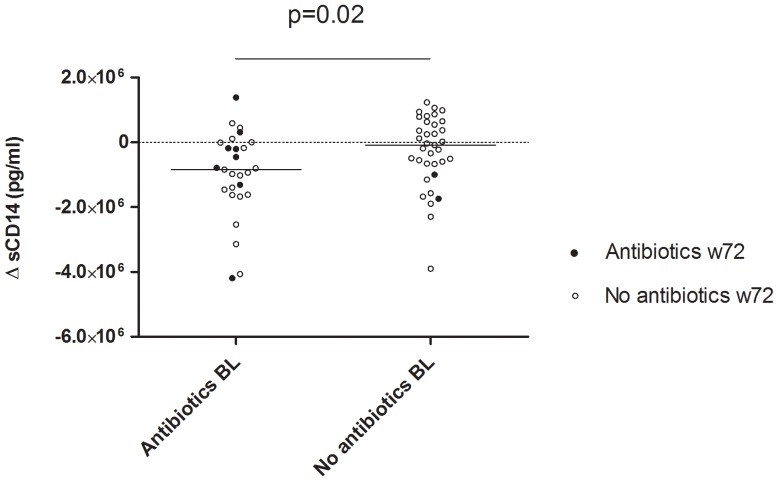
Change (Δ) in sCD14 levels in relation to antibiotics. Change (Δ) in sCD14 levels between baseline (BL) and 72 weeks of treatment (w72). Patients treated with antibiotics at BL had a larger reduction in sCD14 levels at w72 than those who did not receive antibiotics at BL. P-value refers to Mann-Whitney test.

We explored the impact of antibiotics on markers of MT with three comparisons using ANCOVA adjusting for significant covariates. At BL, no difference was found between patients with or without antibiotics. At w72, the levels of sCD14 were lower in patients with ongoing antibiotic treatment vs. those without (Δ = −0.70 µg/ml, p = 0.017). Also, in the untreated group at w72 the patients who had been treated with antibiotics at baseline had lower levels than those who had been untreated at baseline (Δ = −0.47 µg/ml; p = 0.015). Finally, the change in the sCD14 levels between BL and w72 was calculated. A difference between antibiotic treated vs untreated patients at BL was then found (median Δ = −0.85 vs −0.08 µg/ml, p = 0.02) (Figure 7). No effect of antibiotics was observed on the levels of LPS, I-FABP and anti-flagellin antibodies, respectively.

## Discussion

The microbial translocation (MT) markers assessed in our study (LPS, sCD14, I-FABP, anti-flagellin antibodies) were all increased in plasma before treatment and ART influenced the levels during the follow-up period of 72 weeks. The reliability of our results is strengthened by the fact that the patients were included in a randomized clinical trial and were followed closely before, during and after the study. Based on the randomization, we found that the effects on the selected MT parameters could partly differ depending on the type of ART and if antibiotics were given or not. We therefore suggest that other factors than HIV treatment in itself may be relevant for the kinetics of the biomarkers which should be considered when interpreting studies on MT in HIV-1 infected patients.

Data from our RCT cohort showed that the plasma levels of LPS were significantly reduced after 72 weeks of ART with similar pattern in patients treated with NNRTI or PI/r containing therapy. Similar observations on the decrease of plasma LPS after ART have been reported in some [5], [6], [22], but not all studies [23], [24]. Although, estimating MT by measurement of plasma LPS levels is common, both LAL assay variability as well as patient cohort heterogeneity (e.g. diverse rate of opportunistic conditions or hepatitis co-infection) may lie behind the different results. E.g., in a recent work, the kinetics of MT markers (measured by LPS and sCD14 levels) differed according to the severity of pre-ART CD4+ T-cell count [25].

The effect on sCD14 has also been disputed with studies showing decline [23], [26] or increase [22], [24] after initiation of ART. In our study, the sCD14 levels were reduced during ART with a unified pattern in the two treatment arms which is in line with a decreased MT during 72 weeks of ART. It has also been suggested that sCD14 independently predicts mortality in HIV-1 infected patients when adjusting for other key markers [13]. We found a strong inverse correlation between CD4+ T-cells and sCD14 as well as a direct correlation between sCD14 and VL [27]. These findings warrant the hypothesis that higher viral replication is associated with more profound inflammatory response (reflected by sCD14) and leads to lower CD4 T cell counts that also reflect poorer surveillance of gut microbes. The viscous circle of viral replication and inflammation is created and maintained by the “leaky gut” as suggested by Douek et al [28]. One should acknowledge that the sCD14 levels are also elevated in other infections (RSV, Dengue Virus, Mycobacteria) and inflammatory conditions [29], [30]. Thus, although analysis of sCD14 is important for the evaluation of MT in the setting of HIV-1 infected patients, it cannot be considered as a specific marker for MT alone.

Our untreated patients exhibited elevated plasma levels of I-FABP, as compared to historical healthy controls [19], which is in line with the suggestion that I-FABP can be applied as a marker of enterocyte damage and possibly of MT in HIV-1 infected patients [19]. Actually, the levels were similar to those reported in inflammatory bowel disease (IBD) [31], supporting the presence of a significant damage to the enterocytes in HIV-1 patients, at least in those who have a relatively advanced immunodeficiency. To our knowledge, no data on the effect of ART on I-FABP levels have earlier been published. Surprisingly, we found that the I-FABP levels increased in the whole cohort despite 72 weeks of efficient ART. A subgroup analysis revealed that the I-FABP increase occurred in the patients treated with EFV, but not in those with LPV/r. This difference between the two treatment arms could not be explained by any coexisting liver failure, gastrointestinal disease (Inflammatory Bowel Disease, gastroenteritis) or reported side effects such as diarrhea (data not shown). The finding was unexpected particularly in view of the reports claiming a lower level of the residual viremia in EFV-treated patients as compared to those on a PI/r regimen [16], [32], [33]. Although I-FABP has been firmly described as marker of enterocyte damage in other diseases [34], [35], we cannot exclude that the increased I-FABP concentrations in our patients might be related to other events such as drug toxicity or metabolic lipid changes. However, the available laboratory blood and lipid-profile data (baseline and follow up) from our patients did not support these relationships (data not shown). Another hypothesis could be based on that EFV may induce oxidative stress related cell apoptosis [36], [37]. Conversely, PIs are attributed the anti-apoptotic effect [38], [39]. The CD4+ T-cell recovery did not differ significantly between treatment arms, but tended to be higher in LPV/r group. As such, the possibility of immune reconstitution in the gut as a cause of the increasing I-FABP levels in EFV group thus seems to be less likely. Further studies are required to determine if the difference reported by us is related to variation in apoptosis or enhanced turnover of intestinal epithelial cells and as a consequence shedding of I-FABP. In the light of our report, the use of I-FABP for evaluating MT in patients introduced to ART seems questionable, as the kinetics differed compared to the other markers of MT. Most likely the systemic I-FABP levels in patients on ART do not reflect only MT itself. Further studies that include also intestinal biopsies should address these questions.

The bacterial flagellin is known as a microbial compound with strong immune modulatory properties that has an essential role in conditions with intestinal damage, like IBD. Hence, flagellin is regarded as a dominant immune antigen in Crohńs disease (CD) where the antibodies to bacterial flagellin (Anti-CBir1) are detected in about half of patients [40]. Recently we found elevated levels of flagellin-specific IgG in three cohorts of HIV-1 infected patients [11]. Additionally, two years of ART reduced the levels of anti-flagellin IgG, although they were not normalized [20]. In the present study, we confirm and expand our previous observations. Thus, the baseline anti-flagellin levels were increased in all patients and decreased significantly after 72 weeks of ART. However, stratifying the cohort into the treatment arms showed that the significant decrease occurred in the LPV/r containing arm only. This effect was not due to the general polyclonal B-cells activation as the normalization of specific anti-flagellin antibodies to total IgG levels yielded similar results. Our hypothesis is therefore that the decline of the anti-flagellin antibodies was due to decrease of exposure for flagellin itself, as a consequence of restoration of the gut-blood barrier. We also claim that the positive correlation between anti-flagellin antibodies and LPS supports the credibility of anti-flagellin antibodies as a marker of MT, theoretically being more specific than e.g. sCD14, which is a polyclonal marker in nature [41]. The relevance of the observed difference between treatment arms is not clear. It is thus possible, that since both arms tended to decrease the levels of anti-flagellin antibodies, differences in time kinetics between the arms could possibly result in a faster decline in LPV/r group.

Antibiotic treatment leads to changes in the gut microbiota [42], [43]. Also, plasma LPS levels are reduced in SIV infected macaques that are treated with “gut sterilizing” antibiotics [6]. However, so far the impact of antibiotics has not been considered in studies on MT in HIV-1 infected patients. In our study, antibiotic treatment was found to influence the sCD14 levels. ANCOVA-analysis thus revealed that former use of antibiotics at baseline as well as ongoing antibiotic treatment lowered sCD14 levels at week 72 compared to non-antibiotic treated individuals. Additionally, the group using antibiotics at base-line had a larger decrease in sCD14 levels up to week 72. A higher reduction of sCD14 levels in antibiotic treated patients could be due to decreased LPS signaling as a consequence of altered gram-negative intestinal flora. The use of TMP-SMX prophylaxis in ART naïve patients has been associated with a lower annual increase in HIV-1 RNA [44]. Antibiotics like minocycline decrease monocyte activation and can dampen the general inflammation as shown in SIV model [45]. Moreover, rifaximin, a broad-spectrum antibiotic with minimal systematic uptake, has recently been shown to affect endotoxinemia in patients with decompensated cirrhosis. Thus, plasma LPS levels were significantly reduced after 8 weeks course of this antibiotic [46]. These findings together suggest that antibiotic treatment may influence levels of MT markers, potentially providing a bias in studies not compensating for the use.

Collectively, we found that markers of MT were reduced after 72 weeks of ART. EFV and LPV/r based ART, which had similar virological outcome, presented with somewhat different effects on the kinetics of MT markers. Thus, although the profiles of the established markers LPS and sCD14 were unified after 72 weeks of treatment, the levels of I-FABP increased and anti-flagellin antibodies were not significantly reduced, respectively, in the patients on EFV containing therapy. Our data underscore the importance of unraveling the multifaceted correlations between microbial translocation, immune activation, antibiotics usage, HIV-1 replication and the chosen ART. Further longitudinal studies should address this complex issue.

## Supporting Information

Checklist S1 **CONSORT Checklist.**
(DOC)

Protocol S1 **Trial Protocol.**
(DOC)
