# Supplementary material for: Kinetics of Microbial Translocation Markers in Patients on Efavirenz or Lopinavir/r Based Antiretroviral Therapy
Source: PLoS One. 2013 Jan 28;8(1):e55038. doi: 10.1371/journal.pone.0055038 (PMC3557242; doi:10.1371/journal.pone.0055038)
Supplement: Protocol S1 — Trial Protocol. (DOC) [file pone.0055038.s002.doc]

**NORTHiv study**

**A study on ART Naïve patients On different Regimens to Treat Hiv**

**Protocol**

Version of protocol: 3.66

Date for latest up-date: 2005-05-16

Steering committee:

Anders Blaxhult, Stockholm, Sweden

Leo Flamholc, Malmö, Sweden

Magnus Gisslén, Göteborg, Sweden

Vidar Ormaasen, Oslo, Norway

Matti Ristola, Helsinki, Finland

Jussi Sutinen, Helsinki, Finland

Anders Sönnerborg, Huddinge, Sweden

**Table of contents**

1 Study sites and responsible investigators 4

2 Protocol summary 5

2.1 Design 5

2.2 Inclusion 5

2.3 Sample size 5

2.4 Treatment regimens 5

2.5 Primary trial objective 5

2.6 Trial visits 5

2.7 Data collection 5

3 Background and rational 6

3.1 Background and rational 6

3.2 Study drugs 6

3.3 Dosage of study drugs 7

3.4 Interactions with other drugs 7

4 Treatment regimens 7

4.1 Randomised treatment arms 7

4.2 Therapeutic drug monitoring 7

5 Study objectives 7

5.1 Primary objectives 7

5.2 Secondary objectives 8

5.3 Primary endpoint 8

5.4 Secondary endpoints 8

5.5 Definitions for treatment and virological failure 8

6 Investigational plan 9

6.1 Trial design 9

6.2 Trial population 9

6.3 Treatment during the study 10

7 Clinical and laboratory evaluations 10

7.1 Baseline characteristics and evaluations 10

7.2 On trial evaluations 11

8 Adverse events (AE) 12

9 Data analysis and statistical methods 12

10 Roll-over protocol 13

11 Trial administration 13

11.1 Trial monitoring 13

12 Ethical considerations 14

11.1 Regulatory authority and ethics approval 14

11.2 Subject informed consent 14

11.3 Patient confidentiality 14

13 Publications and authorship 14

Table 1 15

Table 2 16

Table 3 17

14 References 18

**1 Study sites and responsible investigators**

See appendix

**2 Protocol summary**

**2.1 Design**

Randomized open label multicentre study.

**2.2 Inclusion**

HIV-1 infected patients naïve to antiretroviral drugs.

No limitations in CD4-cell counts or HIV-RNA levels.

**2.3 Sample size**

Approximately 150-210 patients

**2.4 Treatment regimens**

1. lopinavir/ritonavir (Kaletra®) 400/100 mg BID + 2 NRTIs BID

2. atazanavir (Reyataz ®) 300 mg QD + ritonavir (Norvir®) 100 mg QD + 2 NRTIs QD

3. efavirenz (Stocrin®) 600 mg + QD 2 NRTIs QD

**2.5 Primary trial objective**

1. To determine if there is a difference in the incidence of treatment failure (see 5.5.1 for definition) between the lopinavir/ritonavir BID arm, the atazanavir/ritonavir QD arm, and the efavirenz QD arm.

**2.6 Trial visits**

Pre-entry (earliest 28 days prior treatment initiation) and baseline before therapy start.

On trial evaluations will be completed at week 4 (3-5), 12 (10-14), 24 (22-26), 48 (45-51), 72 (69-75), 96 (93-99), 120 (117-123) and 144 (141-147). In brackets are indicated acceptable deviations from time of follow-up.

On trial evaluation will optionally also be performed at day 7 (6-9), 14 (12-16) and 21 (19-23).

**2.7 Data collection**

A simplified CRF will be filled in and sent by e-mail to the co-ordinating centre following each visit. A copy will be printed out at site and saved in the patient records.

**3 Background and rational**

**3.1 Background and rational**

Antiretroviral combination therapy has markedly decreased the mortality and morbidity in HIV-disease during the last years[1]. The antiretroviral agents presently used are classified in four classes: nucleoside/nucleotide reverse transcriptase inhibitors (NRTIs), non-nucleoside reverse transcriptase inhibitors (NNRTIs), protease inhibitors (PIs), and fusion inhibitors (FIs). An antiretroviral regimen always consists of a combination of drugs, most often three or more from two different classes. An increasing number of new antiretroviral agents have been introduced during the last years and there has been a rapid evolution of new information regarding treatment of HIV. Physicians and patients have to deal with medical associated side effects, problems with adherence, and viral resistance.

The virological goal of the treatment is to suppress and maintain suppression of virological replication, in clinical practice, measured by a decrease in plasma HIV-1 RNA level to below 50 copies/ml. Long-term suppression of HIV-1 RNA levels during antiretroviral treatment depends on good adherence to treatment and achievement of adequate plasma drug levels[2, 3]. In several international studies up to 50% of patients fail treatment with their first antiretroviral combination by the end of one year of therapy. In Scandinavia, higher rates of virological success following antiretroviral therapy have been reported, compared to international studies. Failing therapy is frequently associated with the development of resistance mutations in the HIV genome, often leading to cross-resistance also to other drugs in the same class of antiretrovirals. The chances of a successful treatment are limited if the virus has developed multiple resistance mutations. Consequently, it is important that the first line therapy to the antiretroviral naïve patient is successful. Adherence to the therapy is a major factor to achieve adequate virological efficacy of antiretroviral combination therapy.

To further increase the success rate and effect of treatment, treatment adherence factors such as; treatment regimens with low frequency of side effects, simplified treatment regimens with a low pill burden, few dosing events per day and no food restrictions should be aimed for.

The Swedish guidelines recommend initiation of treatment when the CD4-cell count is in the range between 200 and 350 x 10^6^/l in asymptomatic HIV-infection, and in patients with symptomatic HIV-infection irrespective of the CD4-cell count. As first line therapy, 2 NRTIs in combination with either a NNRTI or a PI is suggested. When PIs are chosen, ritonavir-boosted combinations are most often prefered (PI/r) due to pharmacokinetic and virological reasons[4]. During the last year several drugs have been licensed for dosing once daily (QD), however, only one clinical controlled study between QD and twice-daily (BID) regimens has as yet been published[5]. There are so far no comparable studies between NNRTI-based and PI/r-based regimens given to patients that are antiretroviral naïve published.

The present study aims to compare efficacy, side-effects, and treatment adherence of three different treatment regimens given to antiretroviral naïve HIV-1 infected patients. Treatment will be initiated in accordance with the Swedish National Guidelines. In the BID arm (1), which could be considered as a standard regimen at present, lopinavir/ritonavir, is co-administrated with 2 NRTIs administrated BID. The first QD arm (2) combines the PI atazanavir/ritonavir with 2 NRTIs administrated QD, and in the second QD arm (3), the NNRTI efavirenz is combined with 2 NRTIs administrated QD. All drugs used in the study are licensed in the participating countries and patients will be followed as in clinical practice. Patients could optionally, after informed consent, be followed with additional measurements of HIV-1 RNA after 1, 2, and 3 weeks of treatment to asses the kinetics of viral decline after initiation of treatment.

**3.2 Study drugs**

Lopinavir and efavirenz are approved for first-line treatment of HIV-1 infection. Atazanavir is approved by FDA in USA. In Europe it is available for prescription through licence and assumed to be approved soon. Details of the studied drugs can be found in the product labelling information released by the manufactures.

**3.3 Dosage of study drugs**

Lopinavir is nearly completely metabolised through the hepatic cytochrome P450 system via the CYP3A4 isoform. Lopinavir is, however, co-formulated with a low dose ritonavir (100 mg) which is a potent inhibitor of CYP3A4 and substantially enhances the exposure of lopinavir. The approved dose of lopinavir/ritonavir, 400/100 BID, is used in the study[6]. NNRTIs could induce CYP3A4 and it is recommended to increase the lopinavir/ritonavir dosage to 533/133 mg BID when co-administrated with NNRTIs. Lopinavir/ritonavir is not used together with NNRTIs in this study.

The approved dose of atazanavir is 400 mg QD[7]. Similar to lopinavir, atazanavir is metabolized through CYP3A4 and when co-administrated with ritonavir (100 mg QD), the dosage is normally reduced to 300 mg QD[8]. Tenofovir, a NRTI often used in QD regimens, reduce atazanavir (unboosted) AUC with 25 % and Cmin with 40 % (BMS study AI454-181, unpublished results). When co-administrated with ritonavir-boosted atazanavir (300 mg + 100 mg ritonavir QD), tenofovir reduce AUC with 25 % but due to higher exposure, adequate Cmin (513 ng/ml) is preserved[9]. The Swedish Recommendations favour ritonavir-boosting when PIs are used[4] and by pharmacokinetic and virological reasons atazanavir/ritonavir 300/100 mg QD has been chosen as dosage in this study.

Efavirenz is approved for dosage of 600 mg QD. Therapeutic drug monitoring for dose adjustment is an option and efavirenz plasma levels seems to correlate with virological outcome and central nervous system side effects[10, 11].

**3.4 Interactions with other drugs**

A lot of drug interactions are present between several drugs and the study drugs. The risk of interactions should be carefully considered when other medications will be given concomitant. Since drug interactions with ritonavir-boosted atazanavir are not sufficiently studied, a particular cautious has to be taken with this drug. It should be noted that drugs that decrease the gastric acid, such as H2-antagonists and proton pump inhibitors may substantially reduce the absorption of atazanavir and should thus be avoided.

**4 Treatment regimens**

**4.1 Randomised treatment arms**

Patients will be randomized to one of three study arms (1:1:1). Randomization will be stratified according to CD4-cell count (above or below 200 x 10^6^/l) and plasma HIV-RNA level (above or below 100 000 copies/ml).

Study arms:

1. lopinavir/ritonavir (Kaletra®) 400/100 mg BID + 2 NRTIs BID

2. atazanavir (Reyataz ®) 300 mg QD + ritonavir (Norvir®) 100 mg QD + 2 NRTIs QD

3. efavirenz (Stocrin®) 600 mg QD + 2 NRTIs QD

**4.2 Therapeutic drug monitoring**

Plasma concentrations of lopinavir, atazanavir and efavirenz will be analyzed after 4 weeks of treatment.

The dose of efavirenz may be adjusted after therapeutic drug monitoring in accordance with clinical practice.

**5 Study objectives**

**5.1 Primary objectives**

1. To determine if there is a difference in the incidence of treatment failure (see 5.5.1 for definition) between the lopinavir/ritonavir BID arm, the atazanavir/ritonavir QD arm, and the efavirenz QD arm.

**5.2 Secondary objectives**

1. To clarify whether there are any differences in adherence to treatment between the three arms.

2. To address whether there is a difference between the arms in the incidence of side-effects, including hyperlipidemia, leading to regimen discontinuations or changes.

3. To evaluate differences between the arms in the kinetic of viral suppression during the first four weeks of treatment (optional).

4. To explore the reason for premature discontinuing of randomised treatment.

5. To determine differences in the CD4-cell count response

6. To assess the safety and tolerance of the three treatment arms

7. To assess changes in genotypic resistance in plasma HIV-1 over the study period in the different arms

8. To determine the plasma concentration of the PIs and NNRTIs in steady state, i.e. 2-4 weeks after starting study medication

9. To assess changes in laboratory parameters, plasma lipids included, over the study period

**5.3 Primary endpoint**

1. Incidence of failure (see 5.5.1 for definition).

**5.4 Secondary endpoints**

1. Estimated adherence rate

2. Proportion of treatment discontinuations and changes due to different adverse events.

3. Decrease in HIV-1 RNA during the first 4 weeks of treatment (optional)

4. Proportion of patients with plasma HIV-1 RNA levels below 50 copies/ml after 4, 12, 24, 48, 72, 96, 120 and 144 weeks of treatment. Both Intention to Treat (ITT) and On Treatment (OT) analyses will be performed.

5. Changes in CD4+ T-cell count from baseline.

6. Time to discontinuation or changes of randomised treatment and reasons for this.

7. Incidence of adverse events.

8. Incidence of genotypic resistance to NRTIs, NNRTIs, and PIs that develops during the study

9. Proportion of patients with adjusted drug doses after therapeutic drug monitoring.

10. Correlations between plasma drug concentrations and virological outcome and adverse events.

11. Changes from baseline in plasma lipids.

12. Incidence of hypercholesterolemia and hypertriglyceridemia at baseline and follow-up.

13. Incidence of abnormal laboratory parameters (Hb, LPK, ASAT, ALAT, ALP, Bilirubin, S-amylas, Creatinin)

**5.5 Definitions for treatment and virological failure**

***5.5.1 Treatment failure***

Treatment failure is defined as:

1. Virological failure (see 5.5.2 for definition), or

2. Treatment interruption or change of study treatment due to side-effects (NRTI backbone are allowed to be changed, see 6.3), or

3. Treatment interruption or change of study treatment due to any other reason (NRTI backbone are allowed to be changed, see 6.3), or

4. Missed to follow-up

***5.5.2 Virological failure***

Virological failure is defined as:

1. HIV-1 RNA >50 copies/ml in two consecutive measurements from week 24 and on, or

2. any rise in HIV-1 RNA >50 copies/ml in two consecutive measurements after viral load <50 copies/ml has been reached, or

3. HIV-1 RNA never <50 copies/ml after week 24, or

4. <1 log copies/ml drop in HIV-1 RNA from baseline to week 4 (provided HIV-1 RNA >50 copies/ml), or

5. <2 log copies/ml drop in HIV-1 RNA from baseline to week 12 (provided HIV-1 RNA >50 copies/ml)

**6 Investigational plan**

**6.1 Trial design**

This is a phase IV, randomized open-label multicentre trial on antiretroviral effect of three different treatment regimens, one standard PI/r+2NRTI BID, and two QD regimens, NNRTI+2NRTI and PI/r+2NRTI respectively, on antiretroviral naïve HIV-1 infected patients.

Antiretroviral naïve HIV-1-infected patients with indications to start antiretroviral treatment could be included in the study. If the patient is subject to start treatment and thereby could be included in the study is judged by the clinician in agreement with the patient, and in agreement with the Swedish National Guidelines. An estimation of approximately 50-70 patients in each arm are to be included during two years.

Patients will initiate treatment with one of the three study arms (see 4 Treatment Regimens)

The randomisation is performed at the Department of Infectious Diseases, Sahlgrenska University Hospital/Östra and is stratified for CD4-cell count (< or ≥ 200 x 10^6^/ml) and plasma HIV-1 RNA level (< or ≥ 100 000 copies/ml) (see 7.1.2).

Potentially eligible subjects will attend the clinic for a screening assessment. The CD4-cell count and HIV-1 RNA level at the screening visit will be used in stratification in the randomization process. Subjects will be randomized into one of three treatment arms and followed in accordance with the study plan.

Subjects will continue to receive the randomized treatment for the duration of the trial (i.e. 36 months). The NRTI backbone is allowed to be changed due to reasons other than virological failure, mainly side effects (see 6.3).

For patients in treatment arm 1 that acquire grade 3-4 hyperlipidemia, lopinavir/ritonavir could be switched to atazanavir/ritonavir in a roll-over-protocol (see 9 Roll-over protocol).

The patient could be followed in the study also if a visit has been missed of any reason, however, the baseline visit has to be performed.

The investigational site or the patient can anytime and of any reason decide to discontinue the study.

**6.2 Trial population**

***6.2.1 Inclusion criteria***

A subject will be eligible for inclusion in this trial only if all of the following criteria apply:

1. Male or female, aged 16 years of age.

2. HIV-1 infected as documented by a licensed HIV-1 antibody ELISA.

3. Ability to understand and provide informed consent to participate in the trial.

4. Indication for antiretroviral treatment.

5. Antiretroviral naïve.

6. All clinical laboratory values must be considered not clinically significant – for the potential response to the planned regimen – in the opinion of the investigator.

***6.2.2 Exclusion criteria***

Patients with any of the following criteria are excluded from participation in the study but recorded on a reject log.

1. Subjects being pregnant.

2. Women of childbearing potential not practicing birth control during the study.

3. Subjects with renal failure requiring dialysis.

4. Subjects with concurrent medications which – in the opinion of the investigator and according to drug product labelling – will result in drug interactions with any of the study drugs that are not manageable. Dose adjustments guided by therapeutic drug monitoring are allowed.

5. Resistance to any of the study drugs.

**6.3 Treatment during the study**

The aim is to continue with the randomised treatment throughout the entire study period, i.e. 36 months. Patients must not receive other antiretroviral drugs than those to which the patient has been randomised.

The NRTI backbone is allowed to be changed due to side effects or to other reasons (not virological failure). In that case, the second NRTI backbone should contain of 2 NRTIs, allowed to be dosed either as a QD or a BID regimen, irrespective of what arm the patient has been randomised to. Treatment with the PIs and NNRTI (lopinavir/ritonavir, atazanavir+ritonavir or efavirenz) continues, with the same dosage, as before the change of NRTIs.

Antiviral drugs used to treat other viral infections, such as HSV or CMV, may be used although they might have some additional anti-HIV-effect.

For patients in treatment arm 1 that acquire grade 3-4 hyperlipidemia, lopinavir/ritonavir could be switched to atazanavir/ritonavir in a roll-over-protocol (see 9 Roll-over protocol).

**7 Clinical and laboratory evaluations**

A flow chart showing the timing of trial procedures is shown in table 1 and 2.

Laboratory tests and clinical evaluations are performed as in clinical routine. The compulsory evaluations below are normally included in the clinical routine practice.

**7.1 Baseline characteristics and evaluations**

***7.1.1 Screening and baseline evaluation***

The site must obtain subject informed consent prior to the initiation of the study.

The pre-entry visit for screening must be conducted before, earliest 28 days prior to, the initiation of antiretroviral therapy.

The following evaluations should be performed at pre-entry (screening) visit:

• CD4-cell count

• Quantitative plasma viral load (HIV-1 RNA)

• Hepatitis serology (HBsAg and anti-HCV).

• Weight

CD4-cell count and quantitative HIV-1 RNA PCR obtained in clinical practice before initiation might be used for screening as long as it was obtained within the time limit (≤28 days before treatment initiation at baseline). Hepatitis serology results obtained earlier may be used instead.

The following evaluations should be performed at baseline (day 0):

• Entry criterias and demography including date of birth, gender, race, and mode of transmission of HIV

• CDC HIV classification 1993 version

• Previous HIV-associated conditions, i.e. B and C conditions according to the CDC HIV classification 1993.

• Weight (if not performed at screening)

• Blood lipids, i.e. total cholesterol, LDL, HDL, triglycerids (fasting)

• Hb, LPK, ASAT, ALAT, ALP, Bilirubin, S-amylas, Creatinin, fB-glukos

• Quantitative plasma viral load (HIV-1 RNA)

• 2 x 10 ml plasma for storage in –70^o^C at study site.

***7.1.2 Randomisation***

Patients eligible to the study and who have signed informed consent may be randomised as soon as results from viral load and CD4 cell count measurements are available.

The randomisation (1:1:1) is performed at the Department of Infectious Diseases, Sahlgrenska University Hospital/Östra and is stratified for CD4-cell count (< or ≥ 200 x 10^6^/ml) and plasma HIV-1 RNA level (< or ≥ 100 000 copies/ml).

**7.2 On trial evaluations**

On trial evaluations will be completed at week 4 (3-5), 12 (10-14), 24 (22-26), 48 (45-51), 72 (69-75), 96 (93-99), 120 (117-123) and 144 (141-147). In brackets are indicated acceptable deviations from time of follow-up.

In-between visits according to local praxis, and laboratory tests other than those settled in the study protocol, will be conducted outside the study.

The following evaluations will be performed at each visit if nothing else is stated:

• Current antiretroviral therapy including eventual dose modifications, interruptions, or discontinuations.

• Adherence to treatment

• New HIV-associated complications.

• Side-effects and laboratory pathology

• Weight (week 4, 48, 96, and 144)

• CD4-cell count

• Quantitative plasma viral load (HIV-1 RNA)

• Blood lipids, i.e. total cholesterol, LDL, HDL, triglycerids (fasting)

• Hb, LPK, ASAT, ALAT, ALP, Bilirubin, S-amylas, Creatinin, fB-glukos

• Plasma concentration of NNRTI and PI 12-24 hours after last drug intake (at week 4, 48, 96 and 144)

• 2 x 10 ml plasma for storage in –70^o^C at study site

On trial evaluations will optionally also be performed at day 7 (6-8), 14 (12-16) and 21 (19-23) including:

• Current antiretroviral therapy including eventual dose modifications, interruptions, or discontinuations

• Adherence to treatment

• New HIV-associated complications.

• Side-effects and laboratory pathology

• Quantitative plasma viral load (HIV-1 RNA)

• Hb, LPK, ASAT, ALAT, ALP, Bilirubin, S-amylas, Creatinin, fB-glukos (at day 14)

• 2 x 10 ml plasma for storage in –70^o^C at study site

**8 Adverse events (AE)**

An adverse event (AE) is defined as any untoward medical occurrence in a subject administered a pharmaceutical product which not necessarily have a causal relationship with this treatment. A serious adverse event (SAE) is any AE that is fatal, is life threatening, is disabling or incapaciting, or requires in-patient hospitalisation or prolongs a current hospitalisation.

Any AE occurring during the trial must be documented in the subject’s medical record and on the AE page of the CRF, SAE on the SAE page of the CRF. For each AE recorded the following information is collected: date of onset, date of resolution, description of the event, concomitant medication, test results, and the relationship with one or more of the antiretroviral agents.

All AE that are both serious and unexpected are subject to expedited reporting by the investigator to national regulatory agencies.

If the SAE is fatal or life threatening, a preliminary reporting should be made as soon as possible, but not later than 7 calendar days after first knowledge by the investigator, followed by a complete report within 8 additional calendar days.

For all other serious and unexpected SAEs a report should be made as soon as possible, but not later than 15 calendar days after first knowledge by the investigator.

All AE and SAE should be registered in the CRF and reported to the coordinating center. All SAE should be reported to the coordinating center within the timelines stated above.

The coordinating center will put all AE together and give details in a final report.

**9 Data analysis and statistical methods**

The trial will randomise (1:1:1) 150-210 subjects in three treatment arms.

The subjects will be followed for 144 weeks. Interim analysis will be assessed when all subjects have completed 24, 48 and 96 weeks of therapy.

Both intention to treat analyses and on-treatment analyses will be performed.

The comparisons between treatment arms with respect to treatment failure will be performed by survival tests, which take the follow up periods into account. Other comparisons will be performed by chi-square test (presence of side effects) including all three treatment groups and Kruskal-Wallis test for continuous variables. The last mentioned test is a generalization of Mann-Whitney’s test applicable on three groups. Two-tailed test at the significance level 0.05 will be used.

Figure 1

N=65 in each one of two groups compared. The probability of treatment success is P_1_ and P_2_ in the two groups. From the figure we can read that if P_1_ is 0.70, e.g., then P_2_ has to exceed 0.90 for achieving the power 80%. The line y = x is given in order to facilitate the reading of the figure. The power calculation was performed for Fisher’s exact test at the level 0.05, two-tailed test, though survival tests will be used. However, the power determination for the survival test needs more specifications than available at present and the difference in power is small.

**10 Roll-over protocol**

Patients randomized to and treated in arm 1, lopinavir/ritonavir + 2 NRTIs, can, in case of hyperlipidemia (grade 3-4) change treatment in a roll-over protocol.

Patients could be included in the roll-over protocol if:

1. treated with lopinavir/ritonavir + 2 NRTIs in arm 1, and

2. fasted total Cholesterol >1.6 x upper normal limit or fasted Triglycerides >7.50 g/L in two consecutive measurements

If the patient is eligible to the roll-over protocol, lopinavir/ritonavir (Kaletra®) is changed to atazanavir (Reyataz ®) 300 mg QD + ritonavir (Norvir®) 100 mg QD. The NRTI treatment should be continued.

Follow-up will be performed with the same on-trial evaluation schedule as if they had continued in the original study protocol.

In addition, blood lipids should be measured at the day of treatment change (-7 – 0 days) and also 4 weeks (3-5 weeks) after.

**11 Trial administration**

Simplified case report forms (CRF) will be provided for each subject by the co-ordinating centre. After completion of CRF, the investigator will send a copy of each page by e-mail to the co-ordinating centre after each visit. A copy will be printed and saved in patient records by the investigator at the study site. The data will be entered into a database at the co-ordinating centre.

**11.1 Trial monitoring**

In accordance with applicable regulations, a monitor (study nurse) will periodically contact the site, including conducting on-site visits. The extent, nature and frequency of on-site visits will be based on enrolment rate and quality of the documents provided by the site. During these contacts the monitor will check and assess the progress of the trial,, review trial data collected, conduct source document verification. This will be done in order to verify that the data are authentic, accurate and complete, that safety and rights of subjects are being protected and that the trial is conducted in accordance with the currently approved protocol, good clinical practice and regulatory requirements.

The contacts between the monitor and the sites will be recorded.

**12 Ethical considerations**

**11.1 Regulatory authority and ethics approval**

The study will be review and approved by the Ethics committee and Medical Products Agency (Läkemedelsverket) prior to initiating the study. In accordance with any applicable country-specific regulation, the responsible investigator will obtain approval from the appropriate regulatory agency prior to a site initiating the trial in that country.

The trial will be conducted in accordance with ICH-GCP and all applicable regulations, including, where applicable, the Declaration of Helsinki, June 1964, as modified by the 48^th^ World Medical Association, republic of South Africa, October 1996.

**11.2 Subject informed consent**

The investigator or his/her designee will inform the subject of all aspects pertaining to the subject’s participation in the trial. The process for obtaining subject informed consent will be in accordance with all applicable regulatory requirements. The decision regarding subject participation in the trial that is made by the subject is entirely voluntary. The investigator or his/her designee must emphasise to the subject that consent regarding trial participation may be withdrawn at any time without penalty or loss of benefits to which the subject is otherwise entitled.

**11.3 Patient confidentiality**

The patient identity will only be known by the local sites. CRF’s and other records will be identified by a coded number only to maintain patient confidentiality. All computer entry and networking programs will be done with coded numbers only.

**13 Publications and authorship**

The findings from this trial are intended to be published in peer-reviewed journals. The site responsible investigators decide in agreement whether abstracts are to be submitted to conferences, and how the results are distributed if more than one manuscript is to be drafted.

The trial group as a whole will appear in an appendix in all published manuscripts. Co-authors are selected after a fair evaluation of primarily number of patients entered in to the trial and the level of involvement in the drafting of the manuscript and study protocol. Providing that several manuscripts are to be drafted, a fair rotation among the participating clinical sites of co-authorship slots will be done taking into consideration the number of patients enrolled.

Sponsors will have no influence on contents of the final manuscripts or abstracts.

**Table 1**

Clinical and laboratory evaluations at pre-entry and baseline

| **Evaluation** | **Screening&baseline** |  |
| --- | --- | --- |
|  | **d. -28 - 0** | **d. 0** |
| Informed consent | X |  |
| Entry criteria and demography | X | (X)^f^ |
| Randomisation |  | X^i^ |
| CDC Classification | X | (X)^f^ |
| Medications^a^ | X | X |
| Adherence estimation |  |  |
| HIV-associated conditions^b^ | X | X |
| Plasma HIV-1 RNA quantitative PCR^c^ | X^j^ | X |
| CD4 cell count | X |  |
| Blood lipids (Total Cholesterol, LDL, HDL, TG) (fasting) | X | (X)^f^ |
| Hb, LPK, ASAT, ALAT, ALP, Bilirubin, S-amylas, Creatinin, fB-glukos | X | (X)^f^ |
| Plasma ~~concentration of NNRTI and PI~~^~~d~~^  ~~and~~ 4bOHc^d^ |  | X |
| Plasma sample collection^e^ | X | X |
| Hepatits serology (HBsAg, anti-HCV)^h^ | (X) |  |
| Weight | X | (X)^f^ |

a Antiretroviral therapy including dose modifications, interruptions, discontinuations, and reasons for change should be indicated. All other drugs that are taken regularly should be indicated.

b At baseline, also the history of previous HIV-associated conditions.

c The HIV-1 RNA determination is done real-time at the site using routine virological methodology at that site, preferably the Roche Amplicor Ultrasensitive assay. The same method should be used for the individual subject throughout the trial.

d ~~12-24 hours after the last dose,~~ 10 ml heparin tube, centrifuged, plasma sent to Clin. Pharm., KU Huddinge (label: NORTHiv 4bOHc)

e 2 x 10 ml or 3 x 6 ml EDTA tubes, plasma collected and stored in –70^o^C at study site.

f if not performed on the previous visit

g ~~not necessary if HIV-1 RNA from screening visit performed within 2 weeks from baseline~~

h if not performed earlier

i randomisation could be performed before day 0 provided informed consent is signed and results from viral load and CD4 cell count are available

**j** HIV-1 RNA PCR obtained in clinical practice before initiation might be used for screening as long as it was obtained within the time limit (≤28 days before treatment initiation at baseline)

**Table 2**

Clinical and laboratory evaluations at follow-up

| **Evaluation** | **Follow-up** |  |  |  |  |  |  |  |  |  |  |
| --- | --- | --- | --- | --- | --- | --- | --- | --- | --- | --- | --- |
|  | **d. 7**  **(6-8)**  **optional** | **d. 14**  **(12-16)**  **optional** | **d. 21**  **(19-23)**  **optional** | **w. 4**  **(3-5)** | **w.12**  **(10-14)** | **w.24**  **(22-26)** | **w.48**  **(45-51)** | **w.72**  **(69-75)** | **w.96**  **(93-99)** | **w.120**  **(117-123)** | **w.144**  **(141-147)** |
| CDC Classification |  |  |  | X | X | X | X | X | X | X | X |
| Medications^a^ |  | X |  | X | X | X | X | X | X | X | X |
| Adherence estimation |  | X |  | X | X | X | X | X | X | X | X |
| Adverse events |  | X |  | X | X | X | X | X | X | X | X |
| HIV-associated conditions^b^ |  | X |  | X | X | X | X | X | X | X | X |
| Plasma HIV-1 RNA quantitative PCR^c^ | X | X | X | X | X | X | X | X | X | X | X |
| CD4 cell count |  |  |  | X | X | X | X | X | X | X | X |
| Blood lipids (Total Cholesterol, LDL, HDL, TG) (fasting) |  |  |  | X | X | X | X | X | X | X | X |
| Hb, LPK, ASAT, ALAT, ALP, Bilirubin, S-amylas, Creatinin, fB-glukos |  | X |  | X | X | X | X | X | X | X | X |
| Plasma concentration of NNRTI and PI^d^ |  |  |  | X | (X)^f^ |  | X |  | X |  | X |
| Plasma sample collection^e^ | X | X | X | X | X | X | X | X | X | X | X |
| Weight |  |  |  | X |  |  | X |  | X |  | X |
|  |  |  |  |  |  |  |  |  |  |  |  |

a Antiretroviral therapy including dose modifications, interruptions, discontinuations, and reasons for change should be indicated. All other drugs that are taken regularly should be indicated.

b At baseline, also the history of previous HIV-associated conditions.

c The HIV-1 RNA determination is done real-time at the site using routine virological methodology at that site, preferably the Roche Amplicor Ultrasensitive assay. The same method should be used for the individual subject throughout the trial.

d 10-24 hours after the last dose, 10 ml heparin tube, centrifuged, plasma sent to Clin. Pharm., KU Huddinge

e 2 x 10 ml or 3 x 6 ml EDTA tubes, plasma collected and stored in –70^o^C at study site.

f if not performed on the previous visit

**Table 3**

Roll-over protocol

| **Evaluation** | **Extra visits^a^** |  |
| --- | --- | --- |
|  | **d0**  **(-7-0)** | **w4**  **(3-5)** |
| Blood lipids (Total Cholesterol, LDL, HDL, TG) | X | X |
| Hb, LPK, ASAT, ALAT, ALP, Bilirubin, S-amylas, Creatinin, fB-glukos |  | X |
| Plasma concentration of atazanavir^b^ |  | X |
| Plasma sample collection^c^ | X | X |
|  |  |  |
|  |  |  |

a The day when lopinavir/r is changed to atazanavir+r is regarded as d0 in the roll-over protocol. Besides the extra visits, the patient continues in the follow-up schedule as planned in table 2.

b 10-24 hours after the last dose, 10 ml heparin tube, centrifuged, plasma sent to Clin. Pharm., KU Huddinge

c 2 x 10 ml or 3 x 6 ml EDTA tubes, plasma collected and stored in –70^o^C at study site.

**14 References**

1. Murphy, E.L., et al., *Highly active antiretroviral therapy decreases mortality and morbidity in patients with advanced HIV disease.* Ann Intern Med, 2001. **135**(1): p. 17-26.

2. Casado, J.L., et al., *Percentage of adherence correlates with the risk of protease inhibitor (PI) treatment failure in HIV-infected patients.* Antivir Ther, 1999. **4**(3): p. 157-61.

3. De Vries-Sluijs, T., et al., *Low Nevirapine Plasma Concentrations Predict Virological Failure in an Unselected HIV-1-Infected Population.* Clin Pharmacokinet, 2003. **42**(6): p. 599-605.

4. Sandstrom, E., et al., *Antiretroviral treatment of human immunodeficiency virus infection: Swedish recommendations.* Scand J Infect Dis, 2003. **35**(3): p. 155-67.

5. Maggiolo, F.R., D. Gregis, G. Quinzan, G. Callegaro, A. Arici, C. Ravasio, L. Suter, F., *Once-a-day therapy for HIV infection: a controlled, randomized study in antiretroviral-naive HIV-1-infected patients.* Antiviral Therapy, 2003. **8**(4): p. 339-346.

6. Cvetkovic, R.S. and K.L. Goa, *Lopinavir/ritonavir: a review of its use in the management of HIV infection.* Drugs, 2003. **63**(8): p. 769-802.

7. Sanne, I., et al., *Results of a phase 2 clinical trial at 48 weeks (AI424-007): a dose-ranging, safety, and efficacy comparative trial of atazanavir at three doses in combination with didanosine and stavudine in antiretroviral-naive subjects.* J Acquir Immune Defic Syndr, 2003. **32**(1): p. 18-29.

8. Agarwala, S., R. Russo, and V. Mummaneni. *Steady-state pharmacokinetic (PK) interaction study of atazanavir (atv) and ritonavir (rtv) in healthy subjects (abstract H-1716)*. in *42nd Interscience Conference on Antimicrobial*

*Agents and Chemotherapy (abstract 1689).* 2002. San Diego, USA.

9. Taburet, A.M., C. Piketty, and L. Gerard. *Pharmacokinetic (PK) parameters of atazanavir (atv)/ritonavir(rtv) when combined to tenofovir (tdf) in HIV infected pateints with multiple treatment failures: a substudy of Puzzle2-ANRS 107 trial.* in *10th Conference on Retroviruses and Opportunistic Infections*. 2003. Boston, MA, USA.

10. Langmann, P., et al., *Efavirenz plasma levels for the prediction of treatment failure in heavily pretreated HIV-1 infected patients.* Eur J Med Res, 2002. **7**(7): p. 309-14.

11. Marzolini, C., et al., *Efavirenz plasma levels can predict treatment failure and central nervous system side effects in HIV-1-infected patients.* Aids, 2001. **15**(1): p. 71-5.
